# Help-seeking and challenges faced by transwomen following exposure to gender-based violence; a qualitative study in the Greater Kampala Metropolitan Area, Uganda

**DOI:** 10.1186/s12939-022-01786-2

**Published:** 2022-12-03

**Authors:** Tonny Ssekamatte, Aisha Nalugya, John Bosco Isunju, Muyanga Naume, Patience Oputan, Juliet Kiguli, Solomon Tsebeni Wafula, Simon Peter S. Kibira, David Ssekamatte, Luisa Orza, Richard K. Mugambe, Rhoda K. Wanyenze

**Affiliations:** 1 Department of Disease Control and Environmental Health, Makerere University School of Public Health, New Mulago Gate Rd, Kampala, Uganda; 2 Programs Department, Transgender Equality Uganda, Kampala, Uganda; 3 Department of Community Health and Behavioural Sciences, Makerere University School of Public Health, New Mulago Gate Rd, Kampala, Uganda; 4 Department of Management, Uganda Management Institute, Plot 44-52, Jinja Road, Kampala, Uganda; 5 Frontline AIDS, Brighton, England

**Keywords:** Transwomen, Gender-based violence, HIV/AIDS, Uganda, Stigma

## Abstract

**Background:**

The high prevalence of gender-based violence (GBV) among transwomen is a human rights and public health challenge. Nonetheless, there is limited evidence of sources of GBV support services and the challenges faced by transwomen while help-seeking, especially in transphobic settings like Uganda. This study explored the sources of GBV support services and the challenges faced by transwomen in the Greater Kampala Metropolitan Area during help-seeking.

**Methods:**

A qualitative study design involving 60 transwomen and 10 key informants was conducted. Respondents were recruited using snowball sampling. An in-depth interview (IDI), and a focus group discussion guide were used to collect data from 20 IDI respondents and six focus group discussants. Each focus group discussion averaged six participants. A key informant interview guide was used for key informant interviews. Data were transcribed verbatim and analysed following a thematic framework, informed by the socio-ecological model. Data were organised into themes and subthemes using NVivo 12.0.

**Results:**

The sources of support following exposure to GBV included key population-friendly healthcare facilities and civil society organisations (CSOs), and friends and family. Friends and family provided emotional support while key population-friendly healthcare facilities offered medical services including HIV post-exposure prophylaxis. Key population CSOs provided shelter, nutritional support, and legal advice to GBV victims. Lack of recognition of transgender identity; long distances to healthcare facilities; discrimination by healthcare providers and CSO staff, inappropriate questioning of the trans-gender identity by police officers and healthcare providers, and the lack of trans-competent healthcare providers and legal personnel hindered help-seeking following exposure to GBV.

**Conclusion:**

The immediate sources of GBV support services included key population-friendly healthcare facilities and CSOs, police, and friends and family. However, a significant number of transwomen did not report incidences of GBV. Transwomen were discriminated against at some key population healthcare facilities and CSOs, and police, which hindered help-seeking following exposure to GBV. This study highlights the need to tackle internalized stigma and discrimination against transwomen at the existing sources of GBV support. There is also a need to train law enforcers and legal personnel on the right to access healthcare among transwomen in Uganda.

## Background

Transwomen are at high risk of gender-based violence (GBV), largely due to the social stigma and marginalization associated with not conforming to culturally accepted gender norms [1–3]. GBV refers to the violence that men and women suffer based on their biological sex, gender identity (e.g., transgender), or behaviours that are inconsistent with societal expectations of “being” a man or woman [4, 5]. GBV often results from power inequalities based on gender roles [5]. GBV can take different forms including emotional, physical, economic, and sexual violence orchestrated by different actors in society and deprives an individual of his/her/their human right to have a life free from violence [6].

Although data on GBV among transwomen remains limited in African settings, global statistics show that GBV among transwomen is prevalent and severe [2, 7, 8]. A systematic review by Reisner, Poteat [7] indicated that 34% of transwomen in the United States suffer from sexual violence, 17% from physical violence, and 7% from psychological or emotional violence [7]. In Uganda, GBV is widespread and affects people from different social, economic, and political statuses. According to Uganda's Demographic and Health Survey (UDHS), approximately 51% of cis women aged between 15 and 49 years experience physical violence while 22% suffer from sexual violence compared to only 8% of cis men [9]. Many countries in Africa, including Uganda, have developed strong policy commitments and invested in cis women’s education and empowerment projects to limit GBV, but the outcomes remain dismal [10].

There is a growing body of evidence linking exposure to GBV to other health risks including HIV/sexually transmitted infections (STIs), especially through its intermediate risk factors such as substance use, risky sexual behaviours, limited access to healthcare, and lack of access to justice [11]. The health risks associated with GBV can be mitigated by improving the help-seeking behaviours of survivors [12]. Help-seeking involves a wide range of behaviours such as seeking advice and support from friends and family, obtaining counseling and/or medical care, calling law enforcement in the event of GBV, moving to a violence shelter, pursuing an order of protection, and access to legal services [13]. Help-seeking, however, remains a challenge among transwomen, largely due to combined layers of the stigma associated with transgender identity. Transwomen GBV survivors suffer an additional series of challenges when seeking care including financial barriers, discrimination, lack of trans-competent providers, barriers to health systems, and socioeconomic challenges [14, 15]. Formal support services for survivors in Uganda are inadequate and do not consider the unique needs of transwomen. There are only 13 GBV shelters that provide survivors with temporary refuge and lodging in Uganda [16]), and these are mainly used by cis women. These limitations hinder help-seeking among transwomen.

Failure to seek help following exposure to GBV has negative consequences on the health and well-being of transwomen. It is associated with poor multiple poor health outcomes including HIV infections, mental distress, and premature mortality [5]. Despite these consequences, care and help-seeking and the challenges faced by transwomen following exposure to GBV are not understood [17, 18], especially in transphobic societies like Uganda. Yet, care and help-seeking following GBV are very important for access to emergency services such as medical care, including access to post-exposure prophylaxis for HIV and psychosocial support, and access to legal services. This study, therefore, sought to document the help-seeking and challenges faced by transwomen when seeking related services in the Greater Kampala Metropolitan Area (GKMA), Uganda.

## Materials and methods

### Study setting

The study was carried out in the GKMA which consists of Kampala city and the neighbouring districts of Wakiso, Mukono, Mpigi, Buikwe, and Luweero. It has a rapidly growing population that, in 2019, the Uganda Bureau of Statistics estimated at least six million people in an area of 8,451.9 km 2 (3,263.3 square miles) [19]. It is the major business and industrial hub of Uganda and contributes over 70% of the country's industrial production and over 60% of the country's GDP [20]. Kampala is home to the majority of civil society organizations (CSOs) and key population-friendly healthcare facilities that serve key populations in Uganda, including transwomen.

### Study design and approach

A qualitative study design was used to explore sources of GBV support services and challenges faced by transwomen while help-seeking. A narrative inquiry was used to obtain a thick description of the transwomen’s GBV experiences. The use of narrative inquiry as a qualitative research approach is attracting greater attention due to its ability to aid researchers to uncover real-life experiences through the stories of the respondents [21–24]. The narrative inquiry was adopted because it allows researchers to explore the meanings the study participants attach to GBV experiences, and thus allows amplification of often silent voices [24]. A narrative inquiry has been recommended to understand sensitive issues including stigma, and violence [25], which was the focus of our current study.

### Study population

The study population included transwomen residing in GKMA. The GKMA was chosen as the ideal site for the study since it is home to most transwomen in Uganda. The study included 60 transwomen aged 18 and above. Other respondents included key informants (KIs) such as senior staff of CSOs working with transwomen, representatives of organizations providing access to justice services, trans-friendly sexual and reproductive health (SRH) service providers, and policymakers.

### Sample size, sampling procedure, and data collection techniques

The snowball sampling technique was used in the recruitment of transwomen for the in-depth interviews (IDIs) and focus group discussions (FGDs). CSOs guided the identification of primary seeds for IDIs. The research assistants interviewed the selected seeds and oriented them on how to recruit secondary seeds that fit our criteria. For the IDIs, the selected seeds were required to recruit transgender women who had ever experienced some form of GBV in the last 12 months to understand their experiences while seeking post-GBV support. In contrast, respondents for the FGDs included all transgender women aged 18 years and above, living in the study setting regardless of whether they had experienced any form of GBV. These were interviewed after providing written informed consent. A total of 20 IDIs, six FGDs, and 10 KIs were used to obtain data from the purposively selected respondents in the GKMA. This was informed by the level of theoretical saturation [26]. We purposively interviewed KIs based on their positions and presumed understanding of GBV and referral pathways among transwomen.

Before the FGDs, the research team obtained informed consent from each of the prospective respondents. This was followed by collecting data on their sociodemographic characteristics. For the IDIs, we used a screening tool to obtain a sample of transwomen who had experienced GBV. The FGD and IDI guides first gauged the respondents’ understanding of violence against transwomen (herein referred to as GBV). Afterward, these tools (FGD and IDI guides) guided the discussion on the sources of GBV support services, and challenges faced while help-seeking. Key informant interviews elicited information on how common GBV cases among transwomen were, how the transwomen were treated at the various healthcare facilities while help-seeking following GBV, how the treatment of transwomen influenced help-seeking, sources and availability of GBV support services, and challenges faced by the transwomen while help-seeking.

### Data management and analysis

Qualitative data analysis and presentation were informed by the socio-ecological model [27]. The socio-ecological model can be used to explore the complex interplay between individual, interpersonal, organizational, community, and public policy factors [28–30], and how these shape access and utilization of GBV support services [31]. Before analysis, audio files were transcribed verbatim by three experienced transcribers. As recommended by Creswell and Poth [32], the research team protected the anonymity of the respondents by using unique identification numbers in the data. Additionally, we made use of a data collection matrix to visually locate and identify information relating to the study.

Interviews conducted in the local dialect were transcribed and translated without losing meaning by qualified personnel. We used the interviewee transcript review technique to improve the rigour [33]. This was achieved by sharing and checking interview transcripts with study participants [34]. Upon interviewee transcript review, transcripts were read several times by two members of the study team (TS, MN), who then developed codes and codebook definitions based on the study objectives while integrating emerging themes from the data. The codebook was discussed and agreed upon by all members of the core study team. During the coding phase, the two qualitative data experts measured the inter-coder reliability (ICR) and agreed on how the same data should be coded to ensure the robustness of the coding framework and its application. During the development of the code book and codebook definitions, the qualitative analysts had a 90% agreement on the themes and subthemes. Transcripts were coded using NVivo 12.0 software to ease analysis and ensure validity and credibility. The code reports generated by NVivo 12.0 were read and discussed by study investigators who afterward agreed on themes, organising themes, and basic codes.

### Quality control and assurance

We recruited competent research assistants with experience in working with key populations. These were trained on the study protocol including the data collection tools, sampling procedure, and research ethics. Research assistants were also oriented on the common slang and terminologies used by the trans-women community. All data collection tools were back-translated from English to the local language by two certified translators. Afterward, the original English versions of the data collection tools were compared with the translated versions to ensure consistency of meaning. Research assistants with a good command of English and the local language (Luganda) conducted the interviews. Research assistants were closely supervised during data collection to ensure that they followed the study protocol and adhered to the research ethics.

## Results

### Background characteristics of the respondents

Of the 60 respondents, 33 resided in Mukono district, 56 were aged 18–28 years, 38 had attained secondary education, 55 were single and 18 were Catholic. Thirty-one of the respondents engaged in sex work for a living. All the IDI and FGD respondents in this study had ever experienced some form of GBV (Table 1).

**Table 1:** Background characteristics of the respondents

Variable	Category	Number of respondents
**District of residence**	Mukono	33
	Kampala	21
	Wakiso	6
**Age (years)**	18–28	56
	29–39	4
**Education level**	No formal education	1
	Primary	10
	Secondary	38
	Tertiary	11
**Marital Status**	Single/never married	55
	Married	5
**Religion**	Catholic	18
	Protestant	14
	Muslim	20
	Pentecostal	5
	Seventh-day Adventist	2
	Other	1
**Main source of income**	Sex work	31
	Salaried	9
	Casual work	7
	Other businesses	13
**Type of sex work**	Street-based	2
	Entertainment place-based	7
	Residence/home-based	21
	Other	6
**Living arrangements**	Home with family	14
	Home without family members	21
	Fellow transwomen	14
	No home to stay at	11

### Sources of post GBV support during help-seeking

Respondents mentioned that they sought GBV support from CSOs, healthcare facilities, key population-friendly healthcare facilities, peer educators, and at times family and friends. It was reported that the respondents rarely sought GBV support from the police. The GBV support referral pathways are elaborated in the following subsections.

#### Key population-friendly healthcare facilities

Respondents who experienced GBV reported that they sought support from key population-friendly healthcare facilities and, on rare occasions, public healthcare facilities with key population programs. They often sought post-exposure prophylaxis (PEP) for HIV, HIV testing and counselling services, and treatment for injuries sustained during the violence.

*“Some guy forced me into sex and I got torn. I started bleeding so much and I called my friend for advice. He told me to go to the hospital and explain to the healthcare providers. That is how I first went to facility XXXX. They first gave me an injection and some tablets to reduce pain and afterward I went home.”* (IDI)

*“Besides the healthcare facility, I can’t run anywhere else. Even at the police station, I can’t go there. After going to the facility, I return home and I stay there with my problems until I get better.”* (IDI)

#### Key population-friendly CSOs

Respondents mentioned that they sought GBV support from key population-friendly CSOs. Support received was in form of psychosocial support, e.g., counselling, legal support such as mediation with intimate partners, relocation, shelter, and nutrition support. When asked where they seek GBV support services, one of the respondents said:

*“We always get counseling from YY [transwoman-led key population civil society organization] which helps us. At times, in the event of violence, they give us shelter until we normalize.”* (FGD)

*“When the community where I was living found out I was a transwoman, they beat me up and I ran to PP [key population advocacy civil society organization]. What PP did was to carry out an investigation and when they found out it was true, they relocated me to a safer place.”* (IDI)

*“There are some transwomen who have been evicted from their homes because of transphobia. So, QQ [LGBT organization] provides shelter and feeding to such survivors.”* (FGD)

Respondents reported that key population-friendly CSOs also supported them to access medical services once they experienced GBV. Some of the key population-friendly CSOs also had clinics, from which the GBV survivors obtained emergency healthcare services such as treatment for injuries and counseling.

*“Yes, organizations like YY give us some GBV support. I stole someone’s husband and I was burnt and badly beaten, but I got full treatment under their support.”* (FGD)

*“And then there is ZZZ and AAA [key population-friendly civil society organization]. These have got clinics which can provide healthcare services to transwomen when they experience violence.”* (FGD)

*“We have human rights organizations that we work with. In case you are arrested, they come and stand surety for you. They also mediate with violent partners. These organizations support us to access healthcare services such as counseling.”* (IDI)

Respondents opted not to report instances of GBV to the police but rather to key population CSOs because, compared to the police, these organizations enabled them to access justice. This was because such organizations were managed by paralegals.

*“There are other organizations that have worked hard to save transwomen. Many transwomen report violence such as cases of slapping and beating to these organizations. The executive director of QQ [LGBT organization] together with one of PP [key population advocacy civil society organization] always help out transwomen because they are paralegals.”* (FGD)

*“We don’t report to police because QQ [LGBT organization] has helped us gain justice as transgender people. They work on us and ensure that we get the support needed. Even if at times, justice takes long to be realized.”* (FGD)

#### Police

Only a few of the respondents ever reported instances of GBV to the police. One of the key informants estimated that less than 5% of transwomen did so because the police also perpetrated violence which consequently led to the stigmatization of transwomen. In addition, respondents also pointed out that they feared reporting cases of GBV to police since they did not believe that transwomen's gender identity existed.

*“It is very hard. I think less than 5% of the cases are reported to police because it also stigmatizes transwomen.”* (KI)

*"For police, you can never [report to them]! I have never seen someone courageous enough to go to the police to report the partner or report anyone who has beaten her. Even though it's a landlord who has beaten a transwoman, she cannot go and report it to the police. Usually, the pathway is ‘see the counsellor and then the doctor for medical treatment such as PEP in case you need to.’ But if you mention the word police, they will just say ‘let's leave it!’* (KI)

*"I can't go to the police. They are still in denial that transgender people exist. They say you are a man; how did you get raped!"* (IDI)

Out of all the interviews, only one respondent acknowledged that she reported to the police. The respondent mentioned that the attitude of police officers was now changing compared to the past and that they concentrate on the cases rather than gender identity.

*“Police is now better. Before you would go to the police and the police officers would insult you saying: ‘look at this gay fool.’ But nowadays, if you go to the police, they overlook your gender identity and consider the case that has taken you there."* (IDI)

#### Friends and family

A minority of the respondents mentioned that they sought support from friends and family in the event of exposure to GBV, particularly in healthcare settings. The survivors often confided in friends, especially transwomen. In addition, some friends helped the survivors to access medication in case they were afraid to go to the healthcare facilities.

*“If such a thing [violence] happens, you may run to your friend who is confident and not scared of going to these facilities. She can collect for you the medication if she doesn’t fear.”* (FGD)

*“I have friends who are also transwomen, so I confide in them. I prefer friends from the trans community because they share the same experience so that makes you more comfortable than speaking to another person.”* (IDI)

Whereas a considerable number of respondents sought GBV support from their friends, some felt that some friends would breach confidentiality and spill their secrets.

*“If I need counseling, I have my brothers and sisters-in-law that support me. It’s better than going to your friend and they spill your secrets.”* (FGD)

#### Nowhere to report incidences of GBV

A considerable proportion of the respondents opted not to report GBV experiences since they were not aware of where to go and because their gender identity was not recognized by law.

*“Some of our employers will use all possible ways to entice you into having sex with them. When you refuse, they rape you before you’re out of the office! At times, you don’t want to have sex, but you have nowhere to report such a case.” (FGD)*

*“The law doesn’t favour us [transwomen], so we are not recognized. Police officers take advantage of this and mistreat us. We don’t have anywhere to report them.”* (FGD)

### Challenges faced while seeking GBV support services from the healthcare facility

**Fig. 1:**
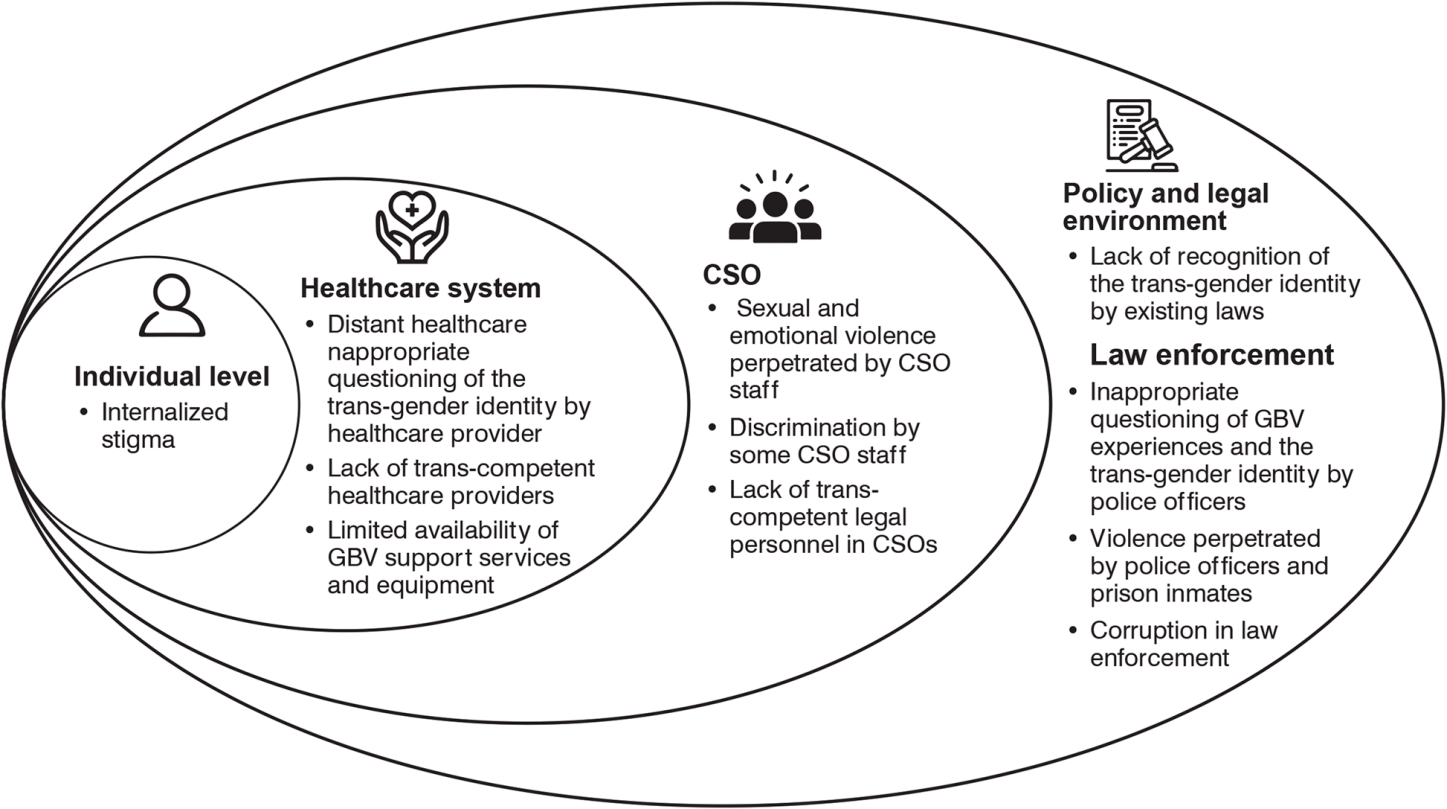
Socio-ecological analysis of challenges faced by transwomen during help-seeking following exposure to GBV

The challenges faced by transwomen while seeking GBV support services were classified as individual, healthcare system, CSO and policy, and legal environment challenges. At an individual level, internalised stigma was common. The healthcare system challenges included distant healthcare facilities, exposure to emotional violence by some healthcare staff, lack of trans-competent healthcare providers, and limited GBV support services and equipment available. The challenges experienced at CSOs included exposure to GBV and discrimination by some staff and a lack of trans-competent legal personnel in CSOs. The policy and legal environment challenges included the criminalisation of same-sex relationships, inappropriate questions about GBV experiences and gender identity, violence by police officers and prison inmates, and corruption by law enforcers (Fig. 1).

### Challenges while help-seeking from healthcare facilities

Whereas healthcare facilities were a haven to some transwomen post-GBV, some study participants reported challenges such as limited health services and equipment, long distances to the facility, abuse from healthcare workers, and gaps in the follow-up of the GBV cases.

### Long distances to healthcare facilities providing GBV support services

Respondents mentioned that healthcare facilities providing key population-friendly services, including GBV support, were located far away from where they lived. They added that due to the long distances to such healthcare facilities and lack of money, it was very difficult for them to travel just after experiencing violence or while sick. The challenge of long distances was exacerbated by a lack of equipment such as the proctology machine in nearby healthcare facilities, which forced many transwomen to travel to the only healthcare facility with such equipment.

*"We would get the treatment but at times the healthcare facility is located far away. For someone that is already very sick, can't even walk, and has not been working, travel to the healthcare facility may become difficult."* (FGD)

*"We have mentioned all those transgender-friendly health facilities but the distance also hinders us from getting GBV services. For instance, only KK [key population-friendly healthcare facility] has the proctology machine. Therefore, if you require such services you have to go to KK. So, we request that they put these machines in other hospitals to reduce travel and related costs."* (FGD)

### Inappropriate questioning of the trans-gender identity by healthcare providers

Respondents reported that the violence they experienced at the hands of healthcare providers while seeking GBV support services discouraged them from visiting healthcare facilities. Specifically, they mentioned inappropriate questions about their gender identity, denial of medical services due to their gender identity, and discrimination and stigma as some of the main challenges they faced while seeking GBV support services. Respondents mentioned that some healthcare providers went on to call the police to get them arrested or media representatives to create a news story.

*"We would go to those healthcare facilities but we are asked a lot of questions. For instance, we are asked questions like ‘what happened to your anus?’ Then they will tell us to lay on the bed for a check-up and by the time you realize, you’re surrounded by police or media personnel.”* (FGD)

Concerning asking inappropriate questions about GBV experience and transwomen's identity, respondents revealed that some healthcare providers asked where and why they had been raped, and how a fellow man can beat them up. To transwomen, such questions were demeaning and traumatizing. This consequently hindered access to and use of available GBV support services in such healthcare facilities.

*“When I was raped, at first I didn’t tell anyone. I was in pain and hurt that I had no one to tell. I felt like killing myself. I used to sit on my bed and shed tears. Until I got someone that I shared my problem with and he took me to the hospital for a checkup. Because of what the doctor took me through, I reached a moment and told them that we should just leave the treatment. The doctor was telling me that, “they can’t rape you! How can they rape you? You also went there when you wanted it* [sexual intercourse]*.” I told the person I came with that we should go.”* (IDI)

*"I have seen some transwomen who have been affected. She is raped, not even raped by one person but gang-raped, and by gang-raped I mean three men and above. You get it. It happens to them. Is she going to go to the hospital? Okay, she is going to go there, and then she is asked ‘where’ she was raped. That question is very rude and demeaning. Do you know what it means? You are raped and when you go to report the case, they ask you ‘where were you raped?’"* (IDI)

*“Usually when you go to access a service such as counselling, HIV testing or consulting and you are telling the story that ‘I was with my boyfriend and we ended up fighting.’ The healthcare provider will ask you ‘how can a fellow man beat you? That’s so weird.’”* (KI)

Respondents revealed that they were at times denied medication once the healthcare providers found out their identity, which made it difficult for them to express themselves while seeking GBV support services.

*"They discriminate against us after knowing who we are. Some can't even give you medication. Because of this, we fear expressing ourselves to them. A health worker might know the cause of let's say bruises in the anus, and they can refuse to give us help.*” (FGD)

### Lack of trans-competent healthcare providers

Some survivors of GBV encountered trans-incompetent healthcare providers. Some healthcare providers in healthcare facilities used by transwomen were not aware of the transgender identity. It was revealed that some healthcare providers failed to distinguish between transwomen and men who have sex with men. It was also revealed that upon knowing that someone was trans, they resorted to discriminating against them or at times preaching to them to rebuke their transgender identity.

*“There is also ignorance about transgender people. When you talk about transwomen, you have to first explain yourself so much. When you talk about GBV, there is no particular service offered or particular point available to take care of transwomen.”* (IDI)

*“I won’t say healthcare providers recognise transwomen because there are still a lot of challenges. They (healthcare providers) don’t know about transwomen, they generalize them as men who have sex with men (MSM). So healthcare providers haven’t learnt to specify who a trans person is and who an MSM is! Most transwomen, when they go to the healthcare facility, they are identified as MSMs. Secondly, even after realizing that one is a transperson, they resort to discriminating against them rather than providing the services they have come for. They are questioned about why they decided to behave a certain way. At times, healthcare providers go on to preach to them, for example, they say, “God loves you”. So, I cannot say we are recognized, and truthfully, we are discriminated against at healthcare facilities. I usually recommend transwomen to go to government facilities but they always decline, because they know, they will not receive the services freely without discriminating against or stigmatizing them” (KI)*

### Limited availability of GBV support services and medical equipment

Besides the lack of competent healthcare providers, respondents also mentioned that healthcare facilities lacked medicines and medical equipment. As a result, GBV survivors were requested to buy such medicines, yet they lacked money. Some respondents mentioned that whereas some healthcare facilities provided GBV services, gaps remained in the follow-up of such GBV cases.

*“In most cases, most of the medications and equipment are not available at the healthcare facilities. They prescribe medication and if you have the money, you go and buy. Yet, in most cases, when you decide to go to these healthcare facilities you don’t have money. So, you just stay without treatment.”* (FGD)

*“The current referral system is not strong. I have sometimes referred transwomen for drugs but they are out of stock or they are not there in the pharmacy.”* (KI)

### Internalized stigma among transwomen

Internalized stigma evidenced by feeling embarrassed to share GBV experiences with healthcare providers hindered transwomen's access to GBV support services. Some transwomen feared being seen by their friends while accessing GBV services from known transgender-friendly healthcare facilities. Respondents also mentioned that they found it difficult to confide in healthcare providers because they believed that they didn't understand them since they are not like them.

*“As a transwoman, you may have a challenge explaining GBV experiences to healthcare providers. If your partner raped you and you have bruises around your anus, explaining what happened to the health worker is challenging and embarrassing, especially to people who don’t understand us.”* (FGD)

*“The reason why some of us get infections is that we fear to go to these healthcare facilities [key population healthcare facilities] since they know us. And once they know you, it might be the beginning point of discrimination.”* (FGD)

### Challenges experienced by transwomen while help-seeking from CSOs

#### Sexual and emotional violence perpetrated by CSO staff

Respondents mentioned that the managers of some non-governmental organizations (NGOs) were also perpetrators of GBV. The transwomen were at times subjected to sexual and emotional violence which in the end discouraged them from seeking GBV support services such as obtaining shelter.

*“Some of the managers of some NGOs are perpetrators of GBV. I decided to come to this NGO and stay. However, I am tortured but I keep quiet. Sometimes he can say ‘if you want to stay here, you must first have sex with me.’ If you refuse, he starts insulting and abusing you and he tells you to leave his shelter. You end up homeless with no one to support you. He doesn’t give me much help but he says hurtful words.”* (IDI)

*“Sometimes you may be hungry but you fear asking the executive director for food because you fear to be harassed and chased out of the shelter. I was in a happy family but the conditions were not good and I left. Sometimes he harasses me telling me I am a failure but I just keep quiet and patiently look for money to get capital and start working again.”* (IDI)

#### Discrimination by some CSO staff

Some respondents experienced discrimination by some CSOs. Preference was often given to men who have sex with men (MSM) as opposed to transwomen.

*"Some organizations start-up to help transwomen and they get funding. However, when we go to these organizations, they discriminate against trans persons and instead attend to MSM."* (FGD)

*"Most of these NGOs help their friends, they used to help us in the past but now that we are many, they select whom to help."* (IDI)

#### Lack of trans-competent legal personnel in CSOs

Respondents mentioned that there was a lack of trans-competent legal personnel in CSOs and that the available legal personnel did not understand their experiences as transwomen.

*“Most legal personnel are straight. They wouldn’t understand us. And they are not in these events or actions with transwomen so they can’t know better.”* (IDI)

### Challenges faced while seeking GBV support services from law enforcement settings

#### Lack of recognition of transgender identity by existing laws

Respondents mentioned that transwomen were not recognized by existing laws and law enforcers such as the police. This meant that they could not express their gender identity while seeking GBV-related services.

*“The law doesn’t recognize transwomen, and police officers are law implementers. You can’t tell them that you are trans yet the trans are not recognized by the law.”* (FGD)

The fact that transwomen were not recognized by the law meant that they often ended up being imprisoned while they sought GBV support services from the police. At times, police officers were also perpetrators of GBV. These challenges are highlighted in the following quotes:

*"Police officers are also perpetrators of GBV. As I said, you might be attacked in the bar and you go to report. When you reach there [the police] and they ask you ‘why did they beat you?’, if you tell the police who you are [transwoman], it might fuel the police officer to put you behind bars."* (FGD)

*“I was arrested by police but I could neither express myself as a woman nor as a man. I was scared of expressing who I am. Sometimes they arrest you for another case and they end up charging you for being a transwoman.”* (IDI)

#### Inappropriate questioning of GBV experiences and trans-gender identity by police officers

Respondents mentioned that they were asked inappropriate questions about their GBV experience and their gender identity whenever they reported a case to the police. Police officers at times asked whether they are female or male, how they got raped, and why they went to a man's place of residence. Transwomen felt embarrassed to tell the police officers that they had sexual intercourse with a man. Consequently, this affected their access to GBV services. This is highlighted in the following quote:

*"When you go to the police to report a case of rape, they will ask you: ‘are you male or female? How were you raped? Why did you go to see the guy in the first place? What were you going to do there?’ How will you tell them that he is your boyfriend and you were going to have sex with him? Will you say it? Of course not! She [transwoman] will stay home and use warm water, salt and painkillers. Maybe she would go for the [HIV] testing after she is better and there are no more bruises. When she is asked how she got infected she wouldn't be able to tell them. How do you even open up that ‘I was raped by five men yet I am a man and I also have a dick." That's what society expects you to be, a man, which you aren't."* (IDI)

#### Violence perpetrated by police officers and prison inmates

Respondents mentioned that they did not have confidence in the police since, at times, this exposed them to more violence. Some respondents mentioned that they had been raped by inmates while in prison cells. In addition, some respondents mentioned that, at times, they had been forced to have unprotected sexual intercourse with police officers with the promise of being released from custody. Some respondents who experienced violence in prisons feared reporting it due to being ashamed of their experience.

*“Some police officers will force you to have sex with them against your will. Some say that ‘I first want to have sex with you and have a feel of how it is before I release you’. They use you without lubricants or a condom and even after sexually abusing you, they end up not releasing you even when you need to test for HIV. You can’t report the abuse anywhere and you don’t even know their HIV status.”* (IDI)

*"Our fellow prisoners rape us. Remember, when the police officer takes you to prison, they will tell everyone ‘that one is gay.’ The prisoners start to applaud the police officer saying ‘ok, bring him in’. They forcefully have sex with you and at times you end up getting injuries. You end up rotting from there and with no treatment in the prison cells. Sometimes, you can't even explain it to the nurses in the prison because they start judging you."* (IDI)

*"When police arrest you, they first call news reporters, parade you, and accuse you of cross-dressing. By morning you are all over the news and that is why we never go to the police. I hate them!"* (IDI)

#### Corruption in law enforcement

It was also reported that access to GBV services offered by police was hindered by the corrupt tendencies of police officers. The respondents mentioned that they often ask them for bribes if they wanted to be helped.

*"Our main referral place is the police, especially for cases of fighting and physical violence. However, at the police, often want kickbacks. Sometimes these police people need bribes, yet sometimes the survivors have no money. In the end, the legal services provided to them [the survivors] are not efficient because of the barriers."* (KI)

*When they arrest us for example for cross-dressing, police officers ask for money especially when you do not have someone to contact, maybe a lawyer or a paralegal. So, you opt to give them money. However, even when you offer the police officers money, they still keep you in their custody” (IDI).*

## Discussion

This study sought to document help-seeking and the challenges faced by transwomen when seeking related services in the GKMA, Uganda. Transwomen sought help from key population-friendly healthcare facilities, key population-friendly CSOs, friends and family, and police in rare cases. The challenges faced by transwomen while seeking GBV-related services included long distances to healthcare facilities; lack of trans-competent healthcare providers and equipment to extend appropriate services to transwomen; GBV experiences at some CSOs; discrimination by some staff at CSOs; and lack of trans-competent legal personnel GBV services.

Our study revealed that transwomen sought help from key population-friendly healthcare facilities for healthcare services such as PEP in the event of exposure to GBV. The majority of transwomen sought PEP from key population healthcare facilities after sexual violence like rape. They also sought treatment for wounds in case of physical violence, and psychosocial support – for example, counseling – once subjected to emotional and mental abuse. Transwomen often opt for key population-friendly healthcare facilities since, at times, they have trans-competent healthcare providers [35] who are more likely to respect their gender identity and confidentiality compared to general healthcare facilities. Such healthcare facilities also have gender-affirming medical services and equipment such as proctoscopes [36], used in examining the rectum, which is not the case in general healthcare facilities. Visiting key population-friendly healthcare facilities makes it easier for transwomen to access other HIV prevention and care services such as condoms, lubricants, and screening for sexually transmitted infections since these healthcare facilities receive and treat them with less discrimination, compared to general healthcare facilities.

Key population-friendly CSOs were seen as a haven for access to GBV services among transwomen. Such organizations create trusted and safe platforms for the provision of psychosocial services such as counselling and legal support. CSOs engage in protecting the human rights of marginalized persons and most at-risk populations such as transwomen through enhanced access to justice, research and advocacy, and legal and human rights awareness. They also have community paralegals who help mediate with intimate partners involved in gender-based violence, while others have been at the forefront of the HIV response with special emphasis on key populations [37]. Some CSOs provide nutritional support and GBV shelters to survivors. GBV shelters are part of the referral pathways and are used to support the provision of temporary refuge, lodging, and other services, and to link survivors to medical, legal, economic, and psychosocial services [16, 37–40]. Transwomen, therefore, opt to turn to key population-friendly CSOs to access related services along the referral pathway.

Our study revealed that some transwomen turned to friends and some family members for psychosocial support once exposed to sexual, physical, and emotional violence. Friends and family were a source of emotional support and reminded survivors to adhere to treatment. Some friends and family members often picked up medication for some GBV survivors. The role of friends and family in increasing access to GBV support services is widely documented [2, 41]. There is evidence of family members protecting transwomen against further victimization from formal resources such as the police [2]. Our findings are consistent with those reported by Nemoto, Bödeker [42] among the transgender population in San Francisco and Oakland, United States. Nemoto, Bödeker [42] reported that some GBV survivors received support from family members and friends.

This study revealed that a minority of GBV survivors reported to the police. The Ugandan police force, through the Department of Child and Family Protection, has the mandate to provide psychosocial support and telephone counseling services to GBV survivors; to link clients in need of care and protection to services or service providers; to follow up reported cases with the area police commanders; and to raise awareness on human rights with the public. A few transwomen in the current study may therefore have opted to seek such services from law enforcement officials, particularly when physical violence erupted. The fact that only a few transwomen sought GBV-related services from the police highlights the disconnect between the trans community and the police, which can be attributed to fear of persecution among transwomen since same-sex intercourse is not legal in Uganda. In addition, many police officers are not aware of transgender identity. Our study also revealed that transwomen did not seek help from the police due to fear of being asked inappropriate questions about their gender identity, and gender-based violence such as rape at the hands of police officers and other inmates. Similar findings have been reported among transwomen in Latin America and the Caribbean [43], Australia [44], Uganda [45, 46], Kenya [47] and South Africa [48, 49]. Our findings, therefore, highlight the need to train law enforcers on transgender identity to increase the uptake of GBV-related services.

The current study revealed that some GBV survivors had nowhere to report such cases. This could be attributed to human rights violations such as the criminalization of transgender identity and same-sex intercourse, religious and cultural beliefs, stigma and discrimination, and violence, as highlighted in Uganda’s penal code [50]. In addition, transwomen do not trust CSOs and law enforcement bodies like the police since they are also perpetrators of GBV. Whereas CSOs and police play an important role in linking GBV survivors to psychosocial support services such as counseling, temporary refuge, and healthcare, managers of some CSOs and police officers were reported to be perpetrators of GBV. Failure to report GBV experiences may also have resulted from the fact that the current existing referral pathway does not take into consideration the unique needs of transwomen. The referral pathway is mainly known and used by cisgender people [5]. The cultural beliefs that portray men as strong enough to defend themselves during GBV exposure may also limit transwomen’s use of the current referral pathways. There is evidence of minimal recognition of transwomen and acknowledgment of their increased risk for HIV infection [51], which can partly explain the non-existence of a GBV referral pathway.

Transwomen in the current study faced the challenge of long distances to key population healthcare facilities while seeking GBV support services such as PEP, HIV testing, and counseling services. Long distances to such facilities led to delayed access to appropriate treatment and increased loss of follow-up if a victim was exposed to HIV. The fact that key population-friendly healthcare facilities were located far away from the residences of transwomen increased the cost of accessing GBV-related services. Many transwomen in Uganda are unemployed and live in absolute poverty. Long distances to healthcare facilities have previously been shown to impede the take-up of healthcare services among transwomen [52].

The lack of trans-competent healthcare providers in both key populations and general healthcare facilities hindered transwomen from accessing GBV-related services. There is evidence of transwomen being prone to disrespect, labeling, breach of confidentiality, discrimination, and stigma at the hands of healthcare providers, which hinders access to GBV services at healthcare facilities. The current study demonstrates the need for health authorities and implementing partners to increase healthcare providers' awareness of transgender identity. This training needs to target healthcare providers in all healthcare facilities irrespective of ownership status (private versus public) and level of service provision. Healthcare providers in key population healthcare facilities should also be trained/retrained since the current study has indicated a knowledge and awareness gap among healthcare providers regarding transgender identity. Training will improve the competence of healthcare providers in the delivery of trans-friendly healthcare services, as has been reported in other studies [43, 52, 53].

The limited scope of GBV services and lack of equipment used for the examination of transwomen hindered access to and use of GBV services. Transwomen suffer from unique injuries and sexually transmitted infections such as inflammation of the rectum, which requires equipment that may not be common in general healthcare facilities, since such conditions are rare in the general population. To the best of our knowledge, only two healthcare facilities (one in the GKMA and the other in western Uganda) currently provide proctology services. This implies that transwomen have a limited choice in terms of access to such services. The limited access to healthcare facilities with the required equipment for examination and treatment of injuries or infections arising from exposure to GBV means that transwomen may find it difficult to access particular treatment and care services when in need. Delayed access to appropriate treatment and care services among transwomen may result in the development of multi-drug resistant pathogens which are more complex to treat [52].

This study revealed that discrimination against transwomen by some CSO staff is a hindrance to access to GBV services. Whereas CSOs are funded to extend services such as HIV testing, lubricants, nutritional support, and legal protection to transwomen, there was an inequality in the provision of such services to transwomen. CSOs favored MSM at the expense of transwomen. Discrimination by CSO staff may have resulted from transphobia, which is often driven by the physical appearance and behavioral dynamics of transwomen [5]. Unlike MSM, transwomen often behave like cis women in terms of their dress code, use of accessories, jewellery, and makeup. This may at times not be considered acceptable to other genders, especially cisgender, thus fueling discrimination [52, 38]. This may have been made worse by the lack of trans-competent legal personnel in CSOs.

### Strengths and limitations

To the best of our knowledge, this is the first study to document help-seeking and challenges faced by transwomen in the process of accessing GBV-related services. The study used a qualitative approach that can enable researchers, policymakers, and implementers to obtain a deeper understanding of GBV among transwomen in transphobic communities. Nonetheless, our study had a few limitations. The study was prone to response bias since the GBV experience is often stigmatizing. Hence, some respondents may have been limited from fully opening up to the research team. However, we trained the research assistants in qualitative skills such as probing to obtain detailed information on the various themes.

## Conclusions

Whereas there was no formal GBV referral pathway, transwomen in the current study used key population-friendly healthcare facilities for medical and psychosocial support, key population-friendly CSOs for shelter and legal support, and friends for emotional support. Only a few of the respondents mentioned approaching the police for GBV support, which was attributed to fear of being asked inappropriate questions about their gender identity, not being recognized by the law, and violence perpetrated by police officers and prison inmates. Some transwomen were not aware of anywhere to report in the event of exposure to GBV. Lack of recognition of transgender identity; long distances to healthcare facilities; exposure to GBV evidenced by discrimination in some healthcare facilities, CSOs, and at the hands of police officers and prison inmates; and the lack of trans-competent healthcare providers and limited scope of GBV services and related equipment made it difficult for transwomen to access GBV support services. Our findings suggest the need to prioritize programs and interventions aimed at reducing GBV toward transwomen and adapting the existing referral pathways to cater to the unique needs of transwomen.

## Data Availability

The transcripts analysed during the current study are available from the corresponding author upon reasonable request.

## Abbreviations

GBV: Gender-based Violence
GKMA: Greater Kampala Metropolitan Area
HIV: Human Immunodeficiency Virus
CSOs: Civil Society Organizations
STIs: Sexually Transmitted Infections
